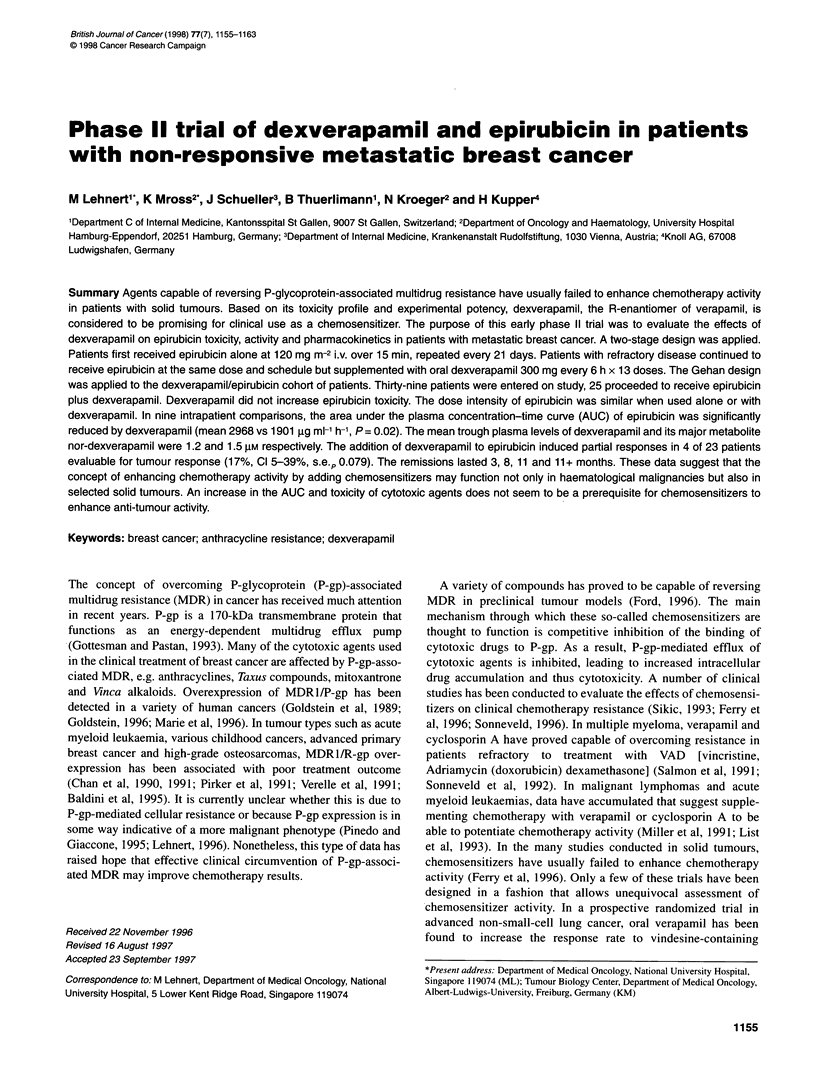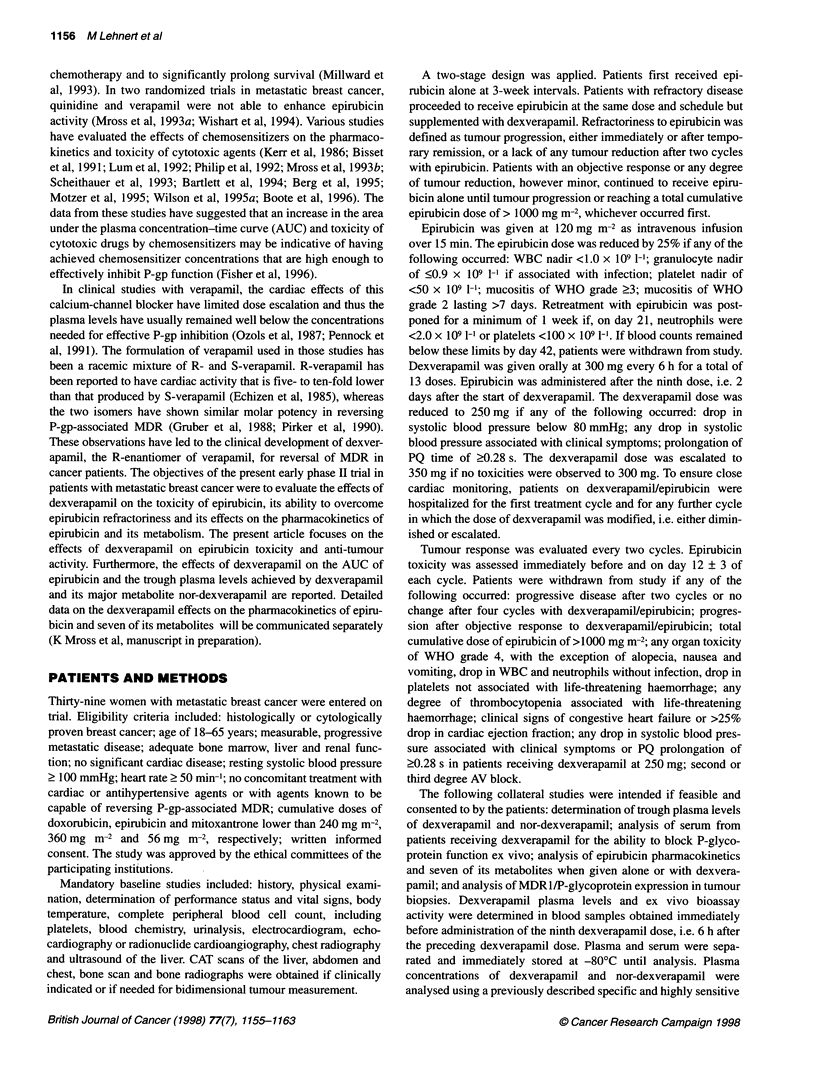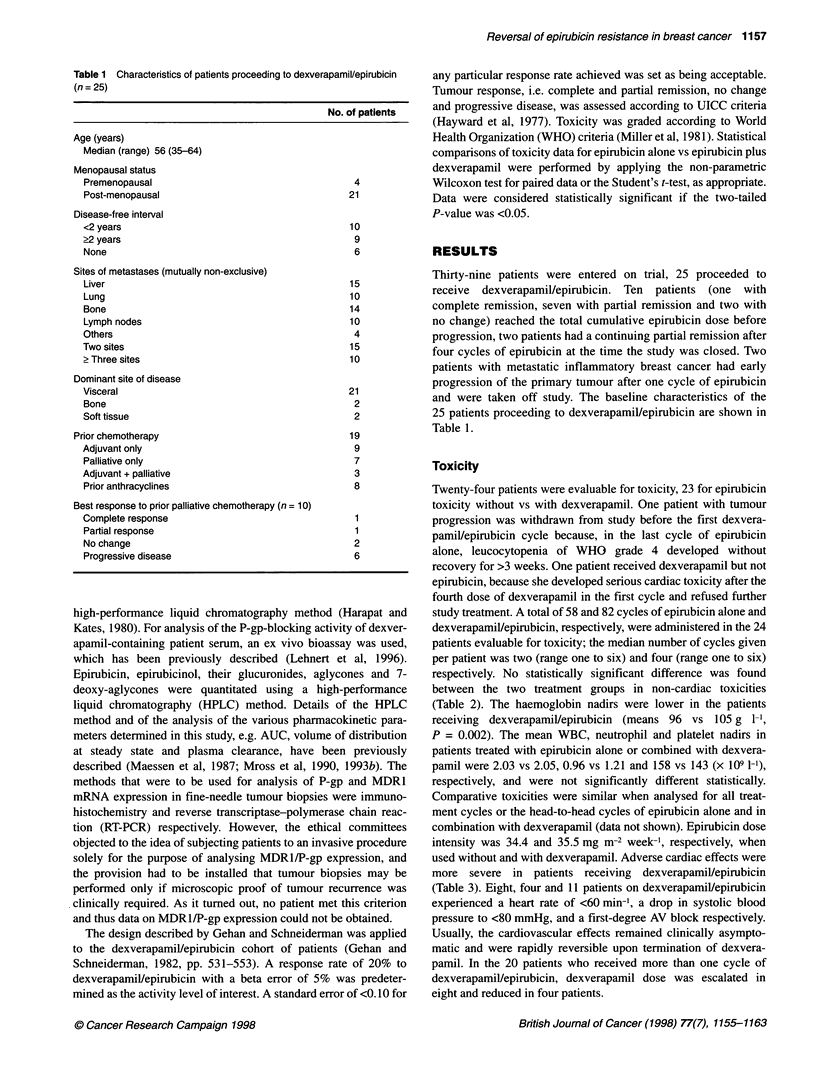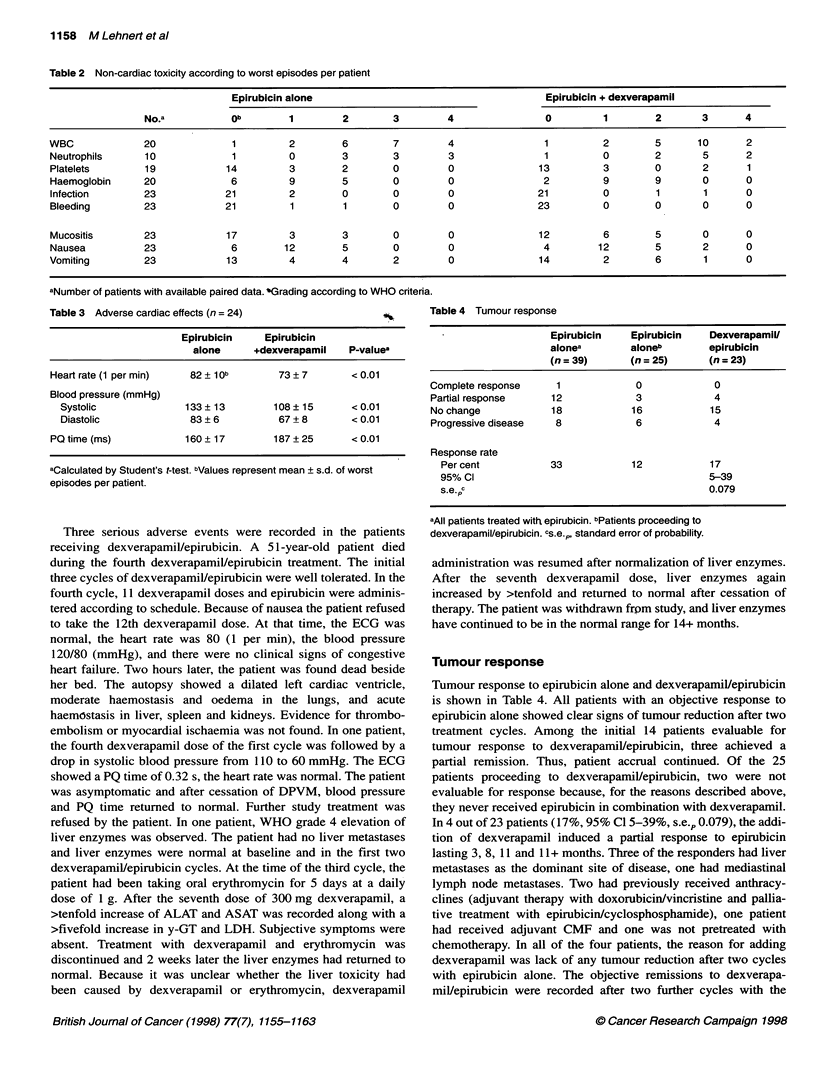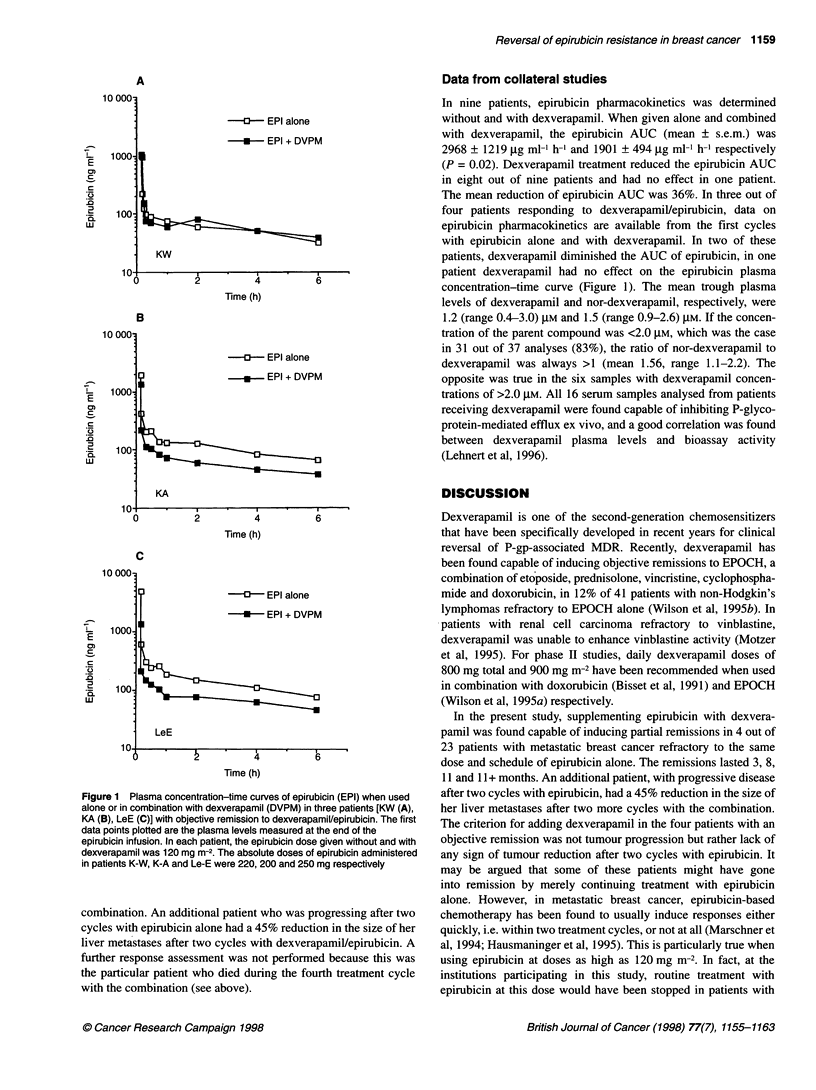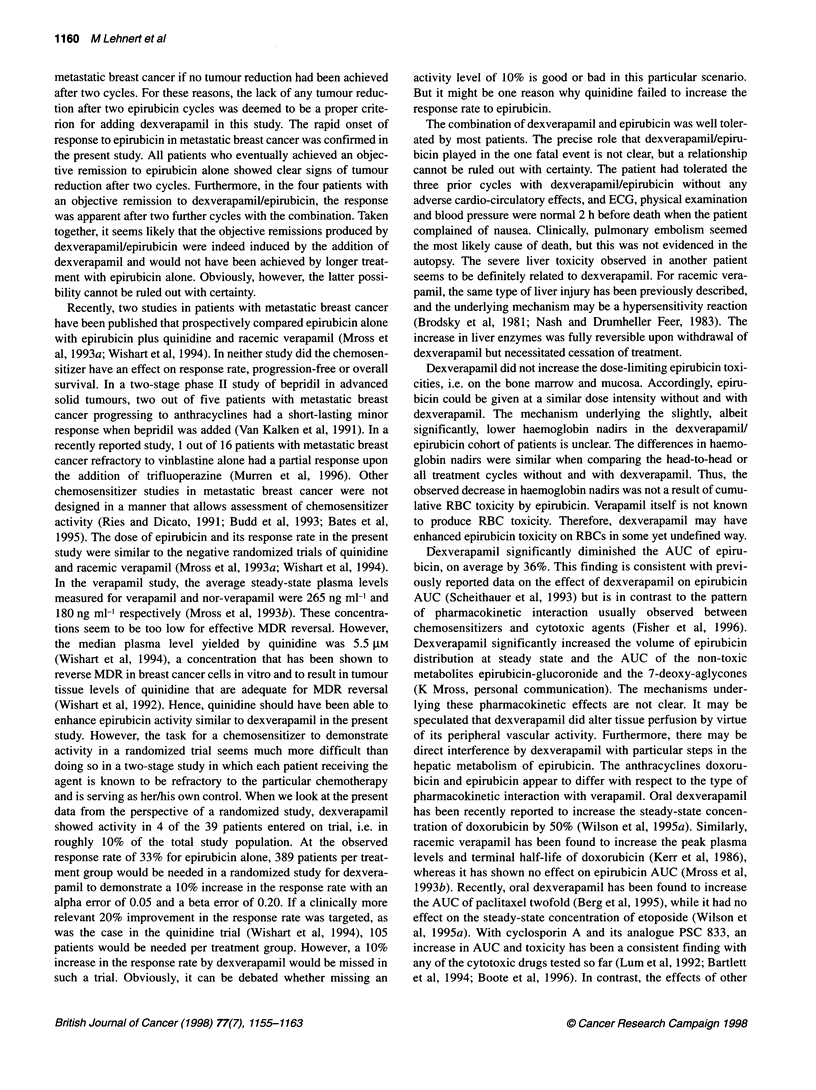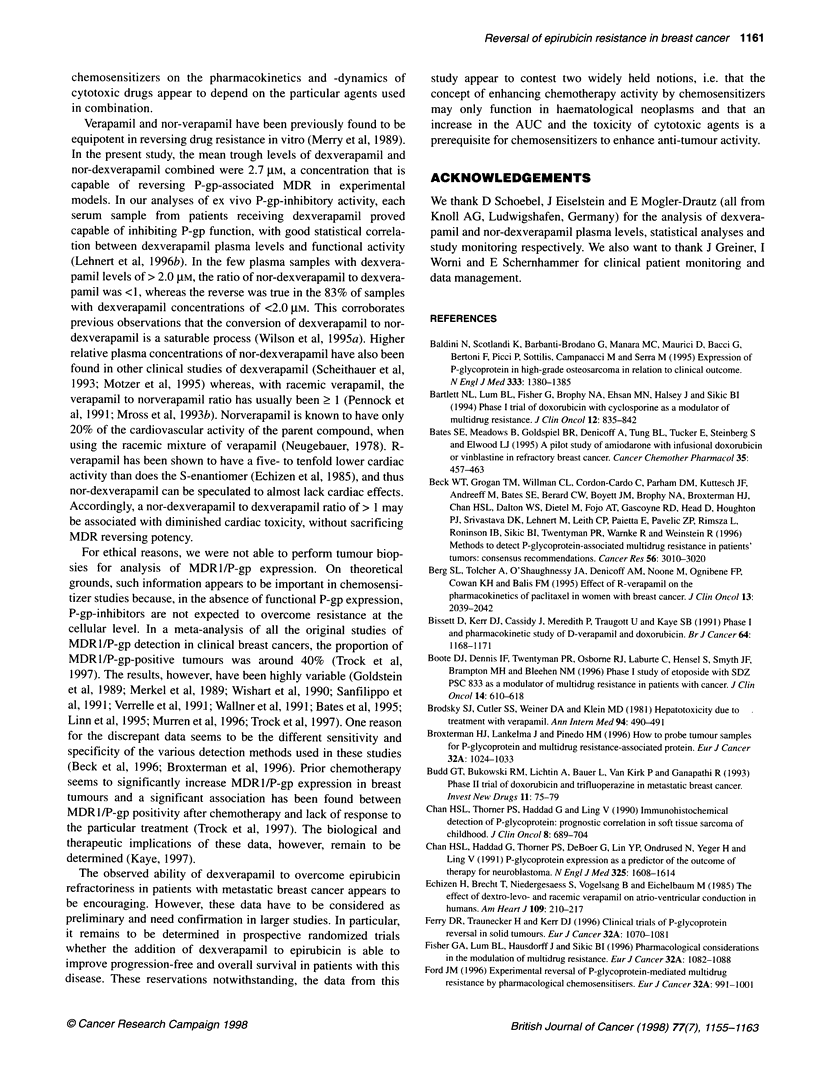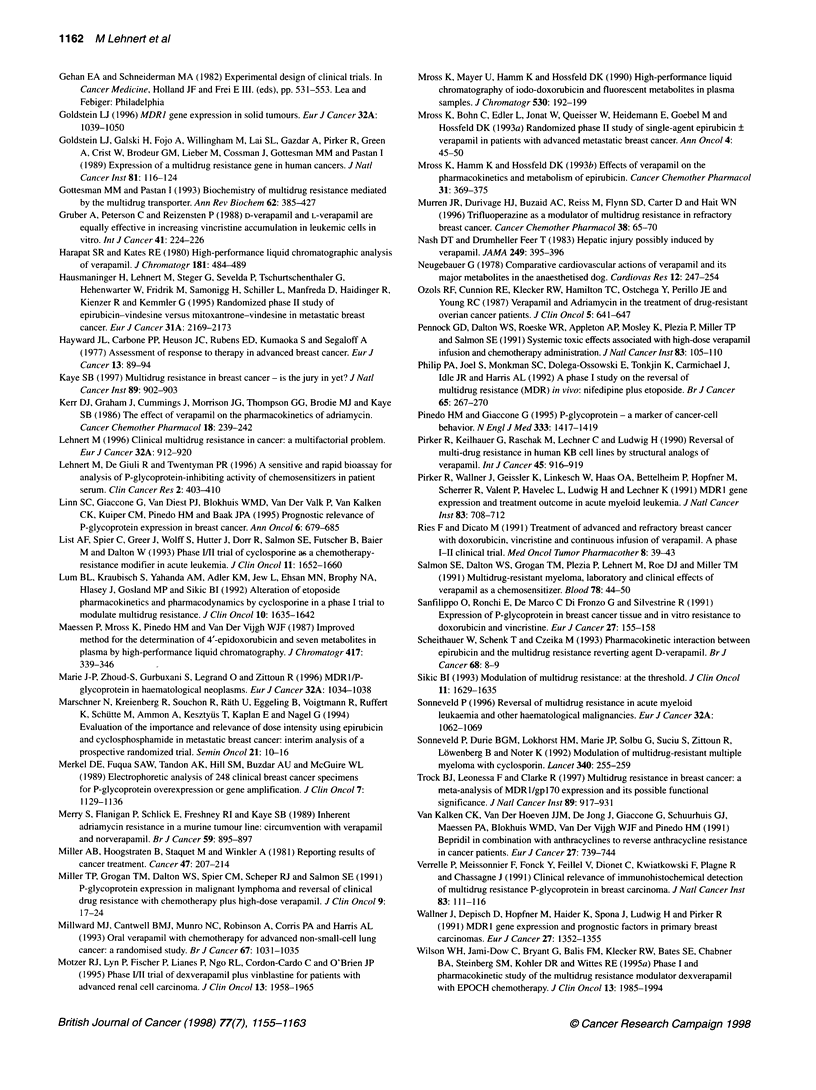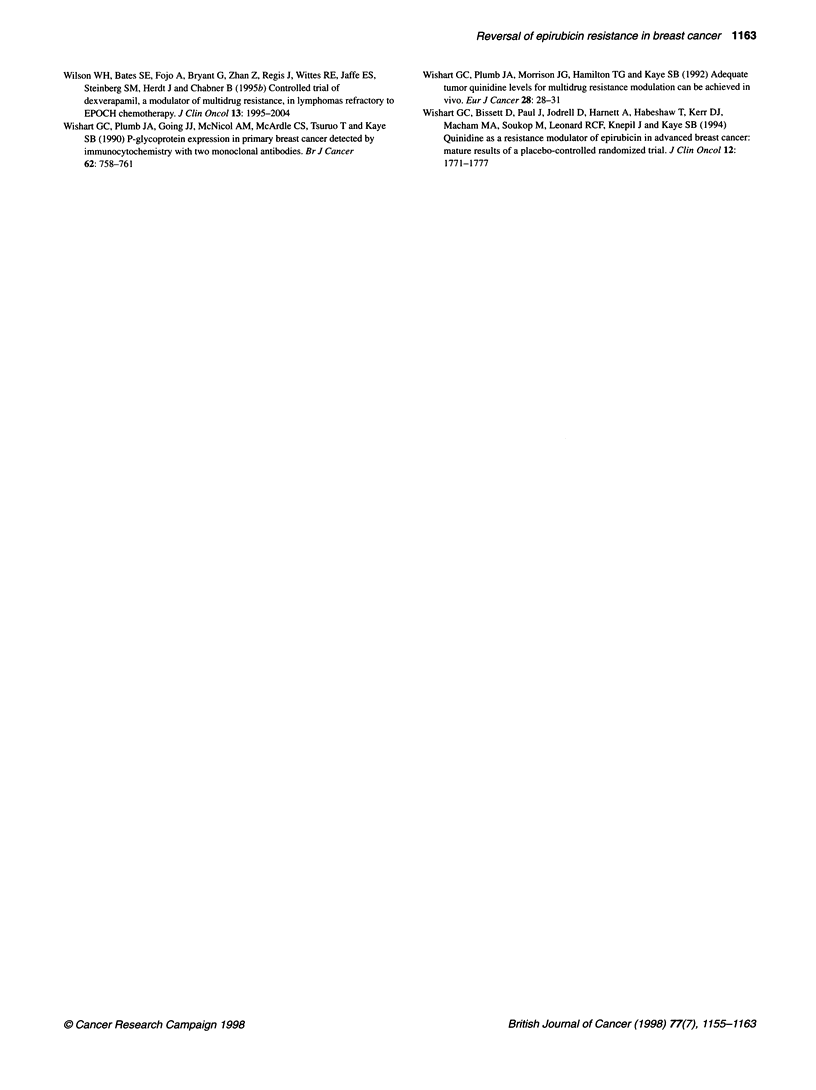# Phase II trial of dexverapamil and epirubicin in patients with non-responsive metastatic breast cancer.

**DOI:** 10.1038/bjc.1998.192

**Published:** 1998-04

**Authors:** M. Lehnert, K. Mross, J. Schueller, B. Thuerlimann, N. Kroeger, H. Kupper

**Affiliations:** Department C of Internal Medicine, Kantonsspital St Gallen, Switzerland.

## Abstract

Agents capable of reversing P-glycoprotein-associated multidrug resistance have usually failed to enhance chemotherapy activity in patients with solid tumours. Based on its toxicity profile and experimental potency, dexverapamil, the R-enantiomer of verapamil, is considered to be promising for clinical use as a chemosensitizer. The purpose of this early phase II trial was to evaluate the effects of dexverapamil on epirubicin toxicity, activity and pharmacokinetics in patients with metastatic breast cancer. A two-stage design was applied. Patients first received epirubicin alone at 120 mg m(-2) i.v. over 15 min, repeated every 21 days. Patients with refractory disease continued to receive epirubicin at the same dose and schedule but supplemented with oral dexverapamil 300 mg every 6 h x 13 doses. The Gehan design was applied to the dexverapamil/epirubicin cohort of patients. Thirty-nine patients were entered on study, 25 proceeded to receive epirubicin plus dexverapamil. Dexverapamil did not increase epirubicin toxicity. The dose intensity of epirubicin was similar when used alone or with dexverapamil. In nine intrapatient comparisons, the area under the plasma concentration-time curve (AUC) of epirubicin was significantly reduced by dexverapamil (mean 2968 vs 1901 microg ml[-1] h[-1], P= 0.02). The mean trough plasma levels of dexverapamil and its major metabolite nor-dexverapamil were 1.2 and 1.5 microM respectively. The addition of dexverapamil to epirubicin induced partial responses in 4 of 23 patients evaluable for tumour response (17%, CI 5-39%, s.e.P 0.079). The remissions lasted 3, 8, 11 and 11+ months. These data suggest that the concept of enhancing chemotherapy activity by adding chemosensitizers may function not only in haematological malignancies but also in selected solid tumours. An increase in the AUC and toxicity of cytotoxic agents does not seem to be a prerequisite for chemosensitizers to enhance anti-tumour activity.


British Journal of Cancer (1998) 77(7), 1155–1163
© 1998 Cancer Research Campaign

### Phase II trial of dexverapamil and epirubicin in patients with non-responsive metastatic breast cancer

**M Lehnert[1*], K Mross[2*], J Schueller[3], B Thuerlimann[1], N Kroeger[2] and H Kupper[4]**

[1]Department C of Internal Medicine, Kantonsspital St Gallen, 9007 St Gallen, Switzerland; [2]Department of Oncology and Haematology, University Hospital Hamburg-Eppendorf, 20251 Hamburg, Germany; [3]Department of Internal Medicine, Krankenanstalt Rudolfstiftung, 1030 Vienna, Austria; [4]Knoll AG, 67008 Ludwigshafen, Germany

**Summary** Agents capable of reversing P-glycoprotein-associated multidrug resistance have usually failed to enhance chemotherapy activity in patients with solid tumours. Based on its toxicity profile and experimental potency, dexverapamil, the R-enantiomer of verapamil, is considered to be promising for clinical use as a chemosensitizer. The purpose of this early phase II trial was to evaluate the effects of dexverapamil on epirubicin toxicity, activity and pharmacokinetics in patients with metastatic breast cancer. A two-stage design was applied. Patients first received epirubicin alone at 120 mg $m^{-2}$ i.v. over 15 min, repeated every 21 days. Patients with refractory disease continued to receive epirubicin at the same dose and schedule but supplemented with oral dexverapamil 300 mg every 6 h × 13 doses. The Gehan design was applied to the dexverapamil/epirubicin cohort of patients. Thirty-nine patients were entered on study, 25 proceeded to receive epirubicin plus dexverapamil. Dexverapamil did not increase epirubicin toxicity. The dose intensity of epirubicin was similar when used alone or with dexverapamil. In nine intrapatient comparisons, the area under the plasma concentration–time curve (AUC) of epirubicin was significantly reduced by dexverapamil (mean 2968 vs 1901 μg $ml^{-1}$ $h^{-1}$, $P = 0.02$). The mean trough plasma levels of dexverapamil and its major metabolite nor-dexverapamil were 1.2 and 1.5 μM respectively. The addition of dexverapamil to epirubicin induced partial responses in 4 of 23 patients evaluable for tumour response (17%, CI 5–39%, s.e.$_p$ 0.079). The remissions lasted 3, 8, 11 and 11+ months. These data suggest that the concept of enhancing chemotherapy activity by adding chemosensitizers may function not only in haematological malignancies but also in selected solid tumours. An increase in the AUC and toxicity of cytotoxic agents does not seem to be a prerequisite for chemosensitizers to enhance anti-tumour activity.

**Keywords:** breast cancer; anthracycline resistance; dexverapamil

The concept of overcoming P-glycoprotein (P-gp)-associated multidrug resistance (MDR) in cancer has received much attention in recent years. P-gp is a 170-kDa transmembrane protein that functions as an energy-dependent multidrug efflux pump (Gottesman and Pastan, 1993). Many of the cytotoxic agents used in the clinical treatment of breast cancer are affected by P-gp-associated MDR, e.g. anthracyclines, *Taxus* compounds, mitoxantrone and *Vinca* alkaloids. Overexpression of MDR1/P-gp has been detected in a variety of human cancers (Goldstein et al, 1989; Goldstein, 1996; Marie et al, 1996). In tumour types such as acute myeloid leukaemia, various childhood cancers, advanced primary breast cancer and high-grade osteosarcomas, MDR1/R-gp overexpression has been associated with poor treatment outcome (Chan et al, 1990, 1991; Pirker et al, 1991; Verelle et al, 1991; Baldini et al, 1995). It is currently unclear whether this is due to P-gp-mediated cellular resistance or because P-gp expression is in some way indicative of a more malignant phenotype (Pinedo and Giaccone, 1995; Lehnert, 1996). Nonetheless, this type of data has raised hope that effective clinical circumvention of P-gp-associated MDR may improve chemotherapy results.

*Received 22 November 1996*
*Revised 16 August 1997*
*Accepted 23 September 1997*

*Correspondence to:* M Lehnert, Department of Medical Oncology, National University Hospital, 5 Lower Kent Ridge Road, Singapore 119074

A variety of compounds has proved to be capable of reversing MDR in preclinical tumour models (Ford, 1996). The main mechanism through which these so-called chemosensitizers are thought to function is competitive inhibition of the binding of cytotoxic drugs to P-gp. As a result, P-gp-mediated efflux of cytotoxic agents is inhibited, leading to increased intracellular drug accumulation and thus cytotoxicity. A number of clinical studies has been conducted to evaluate the effects of chemosensitizers on clinical chemotherapy resistance (Sikic, 1993; Ferry et al, 1996; Sonneveld, 1996). In multiple myeloma, verapamil and cyclosporin A have proved capable of overcoming resistance in patients refractory to treatment with VAD [vincristine, Adriamycin (doxorubicin) dexamethasone] (Salmon et al, 1991; Sonneveld et al, 1992). In malignant lymphomas and acute myeloid leukaemias, data have accumulated that suggest supplementing chemotherapy with verapamil or cyclosporin A to be able to potentiate chemotherapy activity (Miller et al, 1991; List et al, 1993). In the many studies conducted in solid tumours, chemosensitizers have usually failed to enhance chemotherapy activity (Ferry et al, 1996). Only a few of these trials have been designed in a fashion that allows unequivocal assessment of chemosensitizer activity. In a prospective randomized trial in advanced non-small-cell lung cancer, oral verapamil has been found to increase the response rate to vindesine-containing

**Present address:* Department of Medical Oncology, National University Hospital, Singapore 119074 (ML); Tumour Biology Center, Department of Medical Oncology, Albert-Ludwigs-University, Freiburg, Germany (KM)

chemotherapy and to significantly prolong survival (Millward et al, 1993). In two randomized trials in metastatic breast cancer, quinidine and verapamil were not able to enhance epirubicin activity (Mross et al, 1993*a*; Wishart et al, 1994). Various studies have evaluated the effects of chemosensitizers on the pharmacokinetics and toxicity of cytotoxic agents (Kerr et al, 1986; Bisset et al, 1991; Lum et al, 1992; Philip et al, 1992; Mross et al, 1993*b*; Scheithauer et al, 1993; Bartlett et al, 1994; Berg et al, 1995; Motzer et al, 1995; Wilson et al, 1995*a*; Boote et al, 1996). The data from these studies have suggested that an increase in the area under the plasma concentration–time curve (AUC) and toxicity of cytotoxic drugs by chemosensitizers may be indicative of having achieved chemosensitizer concentrations that are high enough to effectively inhibit P-gp function (Fisher et al, 1996).

In clinical studies with verapamil, the cardiac effects of this calcium-channel blocker have limited dose escalation and thus the plasma levels have usually remained well below the concentrations needed for effective P-gp inhibition (Ozols et al, 1987; Pennock et al, 1991). The formulation of verapamil used in those studies has been a racemic mixture of R- and S-verapamil. R-verapamil has been reported to have cardiac activity that is five- to ten-fold lower than that produced by S-verapamil (Echizen et al, 1985), whereas the two isomers have shown similar molar potency in reversing P-gp-associated MDR (Gruber et al, 1988; Pirker et al, 1990). These observations have led to the clinical development of dexverapamil, the R-enantiomer of verapamil, for reversal of MDR in cancer patients. The objectives of the present early phase II trial in patients with metastatic breast cancer were to evaluate the effects of dexverapamil on the toxicity of epirubicin, its ability to overcome epirubicin refractoriness and its effects on the pharmacokinetics of epirubicin and its metabolism. The present article focuses on the effects of dexverapamil on epirubicin toxicity and anti-tumour activity. Furthermore, the effects of dexverapamil on the AUC of epirubicin and the trough plasma levels achieved by dexverapamil and its major metabolite nor-dexverapamil are reported. Detailed data on the dexverapamil effects on the pharmacokinetics of epirubicin and seven of its metabolites will be communicated separately (K Mross et al, manuscript in preparation).

### PATIENTS AND METHODS

Thirty-nine women with metastatic breast cancer were entered on trial. Eligibility criteria included: histologically or cytologically proven breast cancer; age of 18–65 years; measurable, progressive metastatic disease; adequate bone marrow, liver and renal function; no significant cardiac disease; resting systolic blood pressure $\geq$ 100 mmHg; heart rate $\geq$ 50 min$^{-1}$; no concomitant treatment with cardiac or antihypertensive agents or with agents known to be capable of reversing P-gp-associated MDR; cumulative doses of doxorubicin, epirubicin and mitoxantrone lower than 240 mg m$^{-2}$, 360 mg m$^{-2}$ and 56 mg m$^{-2}$, respectively; written informed consent. The study was approved by the ethical committees of the participating institutions.

Mandatory baseline studies included: history, physical examination, determination of performance status and vital signs, body temperature, complete peripheral blood cell count, including platelets, blood chemistry, urinalysis, electrocardiogram, echocardiography or radionuclide cardioangiography, chest radiography and ultrasound of the liver. CAT scans of the liver, abdomen and chest, bone scan and bone radiographs were obtained if clinically indicated or if needed for bidimensional tumour measurement.

A two-stage design was applied. Patients first received epirubicin alone at 3-week intervals. Patients with refractory disease proceeded to receive epirubicin at the same dose and schedule but supplemented with dexverapamil. Refractoriness to epirubicin was defined as tumour progression, either immediately or after temporary remission, or a lack of any tumour reduction after two cycles with epirubicin. Patients with an objective response or any degree of tumour reduction, however minor, continued to receive epirubicin alone until tumour progression or reaching a total cumulative epirubicin dose of > 1000 mg m$^{-2}$, whichever occurred first.

Epirubicin was given at 120 mg m$^{-2}$ as intravenous infusion over 15 min. The epirubicin dose was reduced by 25% if any of the following occurred: WBC nadir $<1.0 \times 10^9$ l$^{-1}$; granulocyte nadir of $\leq 0.9 \times 10^9$ l$^{-1}$ if associated with infection; platelet nadir of $<50 \times 10^9$ l$^{-1}$; mucositis of WHO grade $\geq$3; mucositis of WHO grade 2 lasting >7 days. Retreatment with epirubicin was postponed for a minimum of 1 week if, on day 21, neutrophils were $<2.0 \times 10^9$ l$^{-1}$ or platelets $<100 \times 10^9$ l$^{-1}$. If blood counts remained below these limits by day 42, patients were withdrawn from study. Dexverapamil was given orally at 300 mg every 6 h for a total of 13 doses. Epirubicin was administered after the ninth dose, i.e. 2 days after the start of dexverapamil. The dexverapamil dose was reduced to 250 mg if any of the following occurred: drop in systolic blood pressure below 80 mmHg; any drop in systolic blood pressure associated with clinical symptoms; prolongation of PQ time of $\geq$0.28 s. The dexverapamil dose was escalated to 350 mg if no toxicities were observed to 300 mg. To ensure close cardiac monitoring, patients on dexverapamil/epirubicin were hospitalized for the first treatment cycle and for any further cycle in which the dose of dexverapamil was modified, i.e. either diminished or escalated.

Tumour response was evaluated every two cycles. Epirubicin toxicity was assessed immediately before and on day 12 ± 3 of each cycle. Patients were withdrawn from study if any of the following occurred: progressive disease after two cycles or no change after four cycles with dexverapamil/epirubicin; progression after objective response to dexverapamil/epirubicin; total cumulative dose of epirubicin of >1000 mg m$^{-2}$; any organ toxicity of WHO grade 4, with the exception of alopecia, nausea and vomiting, drop in WBC and neutrophils without infection, drop in platelets not associated with life-threatening haemorrhage; any degree of thrombocytopenia associated with life-threatening haemorrhage; clinical signs of congestive heart failure or >25% drop in cardiac ejection fraction; any drop in systolic blood pressure associated with clinical symptoms or PQ prolongation of $\geq$0.28 s in patients receiving dexverapamil at 250 mg; second or third degree AV block.

The following collateral studies were intended if feasible and consented to by the patients: determination of trough plasma levels of dexverapamil and nor-dexverapamil; analysis of serum from patients receiving dexverapamil for the ability to block P-glycoprotein function ex vivo; analysis of epirubicin pharmacokinetics and seven of its metabolites when given alone or with dexverapamil; and analysis of MDR1/P-glycoprotein expression in tumour biopsies. Dexverapamil plasma levels and ex vivo bioassay activity were determined in blood samples obtained immediately before administration of the ninth dexverapamil dose, i.e. 6 h after the preceding dexverapamil dose. Plasma and serum were separated and immediately stored at –80°C until analysis. Plasma concentrations of dexverapamil and nor-dexverapamil were analysed using a previously described specific and highly sensitive

© Cancer Research Campaign 1998

**Table 1** Characteristics of patients proceeding to dexverapamil/epirubicin (*n* = 25)

| | No. of patients |
|---|---|
| Age (years) | |
| Median (range) 56 (35–64) | |
| Menopausal status | |
| Premenopausal | 4 |
| Post-menopausal | 21 |
| Disease-free interval | |
| <2 years | 10 |
| ≥2 years | 9 |
| None | 6 |
| Sites of metastases (mutually non-exclusive) | |
| Liver | 15 |
| Lung | 10 |
| Bone | 14 |
| Lymph nodes | 10 |
| Others | 4 |
| Two sites | 15 |
| ≥ Three sites | 10 |
| Dominant site of disease | |
| Visceral | 21 |
| Bone | 2 |
| Soft tissue | 2 |
| Prior chemotherapy | 19 |
| Adjuvant only | 9 |
| Palliative only | 7 |
| Adjuvant + palliative | 3 |
| Prior anthracyclines | 8 |
| Best response to prior palliative chemotherapy (*n* = 10) | |
| Complete response | 1 |
| Partial response | 1 |
| No change | 2 |
| Progressive disease | 6 |

high-performance liquid chromatography method (Harapat and Kates, 1980). For analysis of the P-gp-blocking activity of dexverapamil-containing patient serum, an ex vivo bioassay was used, which has been previously described (Lehnert et al, 1996). Epirubicin, epirubicinol, their glucuronides, aglycones and 7-deoxy-aglycones were quantitated using a high-performance liquid chromatography (HPLC) method. Details of the HPLC method and of the analysis of the various pharmacokinetic parameters determined in this study, e.g. AUC, volume of distribution at steady state and plasma clearance, have been previously described (Maessen et al, 1987; Mross et al, 1990, 1993*b*). The methods that were to be used for analysis of P-gp and MDR1 mRNA expression in fine-needle tumour biopsies were immunohistochemistry and reverse transcriptase–polymerase chain reaction (RT-PCR) respectively. However, the ethical committees objected to the idea of subjecting patients to an invasive procedure solely for the purpose of analysing MDR1/P-gp expression, and the provision had to be installed that tumour biopsies may be performed only if microscopic proof of tumour recurrence was clinically required. As it turned out, no patient met this criterion and thus data on MDR1/P-gp expression could not be obtained.

The design described by Gehan and Schneiderman was applied to the dexverapamil/epirubicin cohort of patients (Gehan and Schneiderman, 1982, pp. 531–553). A response rate of 20% to dexverapamil/epirubicin with a beta error of 5% was predetermined as the activity level of interest. A standard error of <0.10 for any particular response rate achieved was set as being acceptable. Tumour response, i.e. complete and partial remission, no change and progressive disease, was assessed according to UICC criteria (Hayward et al, 1977). Toxicity was graded according to World Health Organization (WHO) criteria (Miller et al, 1981). Statistical comparisons of toxicity data for epirubicin alone vs epirubicin plus dexverapamil were performed by applying the non-parametric Wilcoxon test for paired data or the Student's *t*-test, as appropriate. Data were considered statistically significant if the two-tailed *P*-value was <0.05.

### RESULTS

Thirty-nine patients were entered on trial, 25 proceeded to receive dexverapamil/epirubicin. Ten patients (one with complete remission, seven with partial remission and two with no change) reached the total cumulative epirubicin dose before progression, two patients had a continuing partial remission after four cycles of epirubicin at the time the study was closed. Two patients with metastatic inflammatory breast cancer had early progression of the primary tumour after one cycle of epirubicin and were taken off study. The baseline characteristics of the 25 patients proceeding to dexverapamil/epirubicin are shown in Table 1.

### Toxicity

Twenty-four patients were evaluable for toxicity, 23 for epirubicin toxicity without vs with dexverapamil. One patient with tumour progression was withdrawn from study before the first dexverapamil/epirubicin cycle because, in the last cycle of epirubicin alone, leucocytopenia of WHO grade 4 developed without recovery for >3 weeks. One patient received dexverapamil but not epirubicin, because she developed serious cardiac toxicity after the fourth dose of dexverapamil in the first cycle and refused further study treatment. A total of 58 and 82 cycles of epirubicin alone and dexverapamil/epirubicin, respectively, were administered in the 24 patients evaluable for toxicity; the median number of cycles given per patient was two (range one to six) and four (range one to six) respectively. No statistically significant difference was found between the two treatment groups in non-cardiac toxicities (Table 2). The haemoglobin nadirs were lower in the patients receiving dexverapamil/epirubicin (means 96 vs 105 g $l^{-1}$, $P = 0.002$). The mean WBC, neutrophil and platelet nadirs in patients treated with epirubicin alone or combined with dexverapamil were 2.03 vs 2.05, 0.96 vs 1.21 and 158 vs 143 ($\times 10^9$ $l^{-1}$), respectively, and were not significantly different statistically. Comparative toxicities were similar when analysed for all treatment cycles or the head-to-head cycles of epirubicin alone and in combination with dexverapamil (data not shown). Epirubicin dose intensity was 34.4 and 35.5 mg $m^{-2}$ $week^{-1}$, respectively, when used without and with dexverapamil. Adverse cardiac effects were more severe in patients receiving dexverapamil/epirubicin (Table 3). Eight, four and 11 patients on dexverapamil/epirubicin experienced a heart rate of <60 $min^{-1}$, a drop in systolic blood pressure to <80 mmHg, and a first-degree AV block respectively. Usually, the cardiovascular effects remained clinically asymptomatic and were rapidly reversible upon termination of dexverapamil. In the 20 patients who received more than one cycle of dexverapamil/epirubicin, dexverapamil dose was escalated in eight and reduced in four patients.

© Cancer Research Campaign 1998

Table 2 Non-cardiac toxicity according to worst episodes per patient

| | No.[a] | Epirubicin alone | | | | | Epirubicin + dexverapamil | | | | |
|---|---|---|---|---|---|---|---|---|---|---|---|
| | | 0[b] | 1 | 2 | 3 | 4 | 0 | 1 | 2 | 3 | 4 |
| WBC | 20 | 1 | 2 | 6 | 7 | 4 | 1 | 2 | 5 | 10 | 2 |
| Neutrophils | 10 | 1 | 0 | 3 | 3 | 3 | 1 | 0 | 2 | 5 | 2 |
| Platelets | 19 | 14 | 3 | 2 | 0 | 0 | 13 | 3 | 0 | 2 | 1 |
| Haemoglobin | 20 | 6 | 9 | 5 | 0 | 0 | 2 | 9 | 9 | 0 | 0 |
| Infection | 23 | 21 | 2 | 0 | 0 | 0 | 21 | 0 | 1 | 1 | 0 |
| Bleeding | 23 | 21 | 1 | 1 | 0 | 0 | 23 | 0 | 0 | 0 | 0 |
| Mucositis | 23 | 17 | 3 | 3 | 0 | 0 | 12 | 6 | 5 | 0 | 0 |
| Nausea | 23 | 6 | 12 | 5 | 0 | 0 | 4 | 12 | 5 | 2 | 0 |
| Vomiting | 23 | 13 | 4 | 4 | 2 | 0 | 14 | 2 | 6 | 1 | 0 |

[a]Number of patients with available paired data. [b]Grading according to WHO criteria.

Table 3 Adverse cardiac effects ($n$ = 24)

| | Epirubicin alone | Epirubicin +dexverapamil | P-value[a] |
|---|---|---|---|
| Heart rate (1 per min) | 82 ± 10[b] | 73 ± 7 | < 0.01 |
| Blood pressure (mmHg) | | | |
| Systolic | 133 ± 13 | 108 ± 15 | < 0.01 |
| Diastolic | 83 ± 6 | 67 ± 8 | < 0.01 |
| PQ time (ms) | 160 ± 17 | 187 ± 25 | < 0.01 |

[a]Calculated by Student's $t$-test. [b]Values represent mean ± s.d. of worst episodes per patient.

Table 4 Tumour response

| | Epirubicin alone[a] ($n$ = 39) | Epirubicin alone[b] ($n$ = 25) | Dexverapamil/ epirubicin ($n$ = 23) |
|---|---|---|---|
| Complete response | 1 | 0 | 0 |
| Partial response | 12 | 3 | 4 |
| No change | 18 | 16 | 15 |
| Progressive disease | 8 | 6 | 4 |
| Response rate | | | |
| Per cent | 33 | 12 | 17 |
| 95% CI | | | 5–39 |
| s.e.$_p$[c] | | | 0.079 |

[a]All patients treated with epirubicin. [b]Patients proceeding to dexverapamil/epirubicin. [c]s.e.$_p$, standard error of probability.

Three serious adverse events were recorded in the patients receiving dexverapamil/epirubicin. A 51-year-old patient died during the fourth dexverapamil/epirubicin treatment. The initial three cycles of dexverapamil/epirubicin were well tolerated. In the fourth cycle, 11 dexverapamil doses and epirubicin were administered according to schedule. Because of nausea the patient refused to take the 12th dexverapamil dose. At that time, the ECG was normal, the heart rate was 80 (1 per min), the blood pressure 120/80 (mmHg), and there were no clinical signs of congestive heart failure. Two hours later, the patient was found dead beside her bed. The autopsy showed a dilated left cardiac ventricle, moderate haemostasis and oedema in the lungs, and acute haemostasis in liver, spleen and kidneys. Evidence for thromboembolism or myocardial ischaemia was not found. In one patient, the fourth dexverapamil dose of the first cycle was followed by a drop in systolic blood pressure from 110 to 60 mmHg. The ECG showed a PQ time of 0.32 s, the heart rate was normal. The patient was asymptomatic and after cessation of DPVM, blood pressure and PQ time returned to normal. Further study treatment was refused by the patient. In one patient, WHO grade 4 elevation of liver enzymes was observed. The patient had no liver metastases and liver enzymes were normal at baseline and in the first two dexverapamil/epirubicin cycles. At the time of the third cycle, the patient had been taking oral erythromycin for 5 days at a daily dose of 1 g. After the seventh dose of 300 mg dexverapamil, a >tenfold increase of ALAT and ASAT was recorded along with a >fivefold increase in y-GT and LDH. Subjective symptoms were absent. Treatment with dexverapamil and erythromycin was discontinued and 2 weeks later the liver enzymes had returned to normal. Because it was unclear whether the liver toxicity had been caused by dexverapamil or erythromycin, dexverapamil administration was resumed after normalization of liver enzymes. After the seventh dexverapamil dose, liver enzymes again increased by >tenfold and returned to normal after cessation of therapy. The patient was withdrawn from study, and liver enzymes have continued to be in the normal range for 14+ months.

### Tumour response

Tumour response to epirubicin alone and dexverapamil/epirubicin is shown in Table 4. All patients with an objective response to epirubicin alone showed clear signs of tumour reduction after two treatment cycles. Among the initial 14 patients evaluable for tumour response to dexverapamil/epirubicin, three achieved a partial remission. Thus, patient accrual continued. Of the 25 patients proceeding to dexverapamil/epirubicin, two were not evaluable for response because, for the reasons described above, they never received epirubicin in combination with dexverapamil. In 4 out of 23 patients (17%, 95% Cl 5–39%, s.e.$_p$ 0.079), the addition of dexverapamil induced a partial response to epirubicin lasting 3, 8, 11 and 11+ months. Three of the responders had liver metastases as the dominant site of disease, one had mediastinal lymph node metastases. Two had previously received anthracyclines (adjuvant therapy with doxorubicin/vincristine and palliative treatment with epirubicin/cyclosphosphamide), one patient had received adjuvant CMF and one was not pretreated with chemotherapy. In all of the four patients, the reason for adding dexverapamil was lack of any tumour reduction after two cycles with epirubicin alone. The objective remissions to dexverapamil/epirubicin were recorded after two further cycles with the

© Cancer Research Campaign 1998

**Figure 1** Plasma concentration–time curves of epirubicin (EPI) when used alone or in combination with dexverapamil (DVPM) in three patients [KW (**A**), KA (**B**), LeE (**C**)] with objective remission to dexverapamil/epirubicin. The first data points plotted are the plasma levels measured at the end of the epirubicin infusion. In each patient, the epirubicin dose given without and with dexverapamil was 120 mg $m^{-2}$. The absolute doses of epirubicin administered in patients K-W, K-A and Le-E were 220, 200 and 250 mg respectively combination. An additional patient who was progressing after two cycles with epirubicin alone had a 45% reduction in the size of her liver metastases after two cycles with dexverapamil/epirubicin. A further response assessment was not performed because this was the particular patient who died during the fourth treatment cycle with the combination (see above).

### Data from collateral studies

In nine patients, epirubicin pharmacokinetics was determined without and with dexverapamil. When given alone and combined with dexverapamil, the epirubicin AUC (mean ± s.e.m.) was 2968 ± 1219 μg $ml^{-1}$ $h^{-1}$ and 1901 ± 494 μg $ml^{-1}$ $h^{-1}$ respectively ($P$ = 0.02). Dexverapamil treatment reduced the epirubicin AUC in eight out of nine patients and had no effect in one patient. The mean reduction of epirubicin AUC was 36%. In three out of four patients responding to dexverapamil/epirubicin, data on epirubicin pharmacokinetics are available from the first cycles with epirubicin alone and with dexverapamil. In two of these patients, dexverapamil diminished the AUC of epirubicin, in one patient dexverapamil had no effect on the epirubicin plasma concentration–time curve (Figure 1). The mean trough plasma levels of dexverapamil and nor-dexverapamil, respectively, were 1.2 (range 0.4–3.0) μM and 1.5 (range 0.9–2.6) μM. If the concentration of the parent compound was <2.0 μM, which was the case in 31 out of 37 analyses (83%), the ratio of nor-dexverapamil to dexverapamil was always >1 (mean 1.56, range 1.1–2.2). The opposite was true in the six samples with dexverapamil concentrations of >2.0 μM. All 16 serum samples analysed from patients receiving dexverapamil were found capable of inhibiting P-glycoprotein-mediated efflux ex vivo, and a good correlation was found between dexverapamil plasma levels and bioassay activity (Lehnert et al, 1996).

### DISCUSSION

Dexverapamil is one of the second-generation chemosensitizers that have been specifically developed in recent years for clinical reversal of P-gp-associated MDR. Recently, dexverapamil has been found capable of inducing objective remissions to EPOCH, a combination of etoposide, prednisolone, vincristine, cyclophosphamide and doxorubicin, in 12% of 41 patients with non-Hodgkin's lymphomas refractory to EPOCH alone (Wilson et al, 1995*b*). In patients with renal cell carcinoma refractory to vinblastine, dexverapamil was unable to enhance vinblastine activity (Motzer et al, 1995). For phase II studies, daily dexverapamil doses of 800 mg total and 900 mg $m^{-2}$ have been recommended when used in combination with doxorubicin (Bisset et al, 1991) and EPOCH (Wilson et al, 1995*a*) respectively.

In the present study, supplementing epirubicin with dexverapamil was found capable of inducing partial remissions in 4 out of 23 patients with metastatic breast cancer refractory to the same dose and schedule of epirubicin alone. The remissions lasted 3, 8, 11 and 11+ months. An additional patient, with progressive disease after two cycles with epirubicin, had a 45% reduction in the size of her liver metastases after two more cycles with the combination. The criterion for adding dexverapamil in the four patients with an objective remission was not tumour progression but rather lack of any sign of tumour reduction after two cycles with epirubicin. It may be argued that some of these patients might have gone into remission by merely continuing treatment with epirubicin alone. However, in metastatic breast cancer, epirubicin-based chemotherapy has been found to usually induce responses either quickly, i.e. within two treatment cycles, or not at all (Marschner et al, 1994; Hausmaninger et al, 1995). This is particularly true when using epirubicin at doses as high as 120 mg $m^{-2}$. In fact, at the institutions participating in this study, routine treatment with epirubicin at this dose would have been stopped in patients with

© *Cancer Research Campaign 1998* 

metastatic breast cancer if no tumour reduction had been achieved after two cycles. For these reasons, the lack of any tumour reduction after two epirubicin cycles was deemed to be a proper criterion for adding dexverapamil in this study. The rapid onset of response to epirubicin in metastatic breast cancer was confirmed in the present study. All patients who eventually achieved an objective remission to epirubicin alone showed clear signs of tumour reduction after two cycles. Furthermore, in the four patients with an objective remission to dexverapamil/epirubicin, the response was apparent after two further cycles with the combination. Taken together, it seems likely that the objective remissions produced by dexverapamil/epirubicin were indeed induced by the addition of dexverapamil and would not have been achieved by longer treatment with epirubicin alone. Obviously, however, the latter possibility cannot be ruled out with certainty.

Recently, two studies in patients with metastatic breast cancer have been published that prospectively compared epirubicin alone with epirubicin plus quinidine and racemic verapamil (Mross et al, 1993*a*; Wishart et al, 1994). In neither study did the chemosensitizer have an effect on response rate, progression-free or overall survival. In a two-stage phase II study of bepridil in advanced solid tumours, two out of five patients with metastatic breast cancer progressing to anthracyclines had a short-lasting minor response when bepridil was added (Van Kalken et al, 1991). In a recently reported study, 1 out of 16 patients with metastatic breast cancer refractory to vinblastine alone had a partial response upon the addition of trifluoperazine (Murren et al, 1996). Other chemosensitizer studies in metastatic breast cancer were not designed in a manner that allows assessment of chemosensitizer activity (Ries and Dicato, 1991; Budd et al, 1993; Bates et al, 1995). The dose of epirubicin and its response rate in the present study were similar to the negative randomized trials of quinidine and racemic verapamil (Mross et al, 1993*a*; Wishart et al, 1994). In the verapamil study, the average steady-state plasma levels measured for verapamil and nor-verapamil were 265 ng $ml^{-1}$ and 180 ng $ml^{-1}$ respectively (Mross et al, 1993*b*). These concentrations seem to be too low for effective MDR reversal. However, the median plasma level yielded by quinidine was 5.5 $\mu$M (Wishart et al, 1994), a concentration that has been shown to reverse MDR in breast cancer cells in vitro and to result in tumour tissue levels of quinidine that are adequate for MDR reversal (Wishart et al, 1992). Hence, quinidine should have been able to enhance epirubicin activity similar to dexverapamil in the present study. However, the task for a chemosensitizer to demonstrate activity in a randomized trial seems much more difficult than doing so in a two-stage study in which each patient receiving the agent is known to be refractory to the particular chemotherapy and is serving as her/his own control. When we look at the present data from the perspective of a randomized study, dexverapamil showed activity in 4 of the 39 patients entered on trial, i.e. in roughly 10% of the total study population. At the observed response rate of 33% for epirubicin alone, 389 patients per treatment group would be needed in a randomized study for dexverapamil to demonstrate a 10% increase in the response rate with an alpha error of 0.05 and a beta error of 0.20. If a clinically more relevant 20% improvement in the response rate was targeted, as was the case in the quinidine trial (Wishart et al, 1994), 105 patients would be needed per treatment group. However, a 10% increase in the response rate by dexverapamil would be missed in such a trial. Obviously, it can be debated whether missing an activity level of 10% is good or bad in this particular scenario. But it might be one reason why quinidine failed to increase the response rate to epirubicin.

The combination of dexverapamil and epirubicin was well tolerated by most patients. The precise role that dexverapamil/epirubicin played in the one fatal event is not clear, but a relationship cannot be ruled out with certainty. The patient had tolerated the three prior cycles with dexverapamil/epirubicin without any adverse cardio-circulatory effects, and ECG, physical examination and blood pressure were normal 2 h before death when the patient complained of nausea. Clinically, pulmonary embolism seemed the most likely cause of death, but this was not evidenced in the autopsy. The severe liver toxicity observed in another patient seems to be definitely related to dexverapamil. For racemic verapamil, the same type of liver injury has been previously described, and the underlying mechanism may be a hypersensitivity reaction (Brodsky et al, 1981; Nash and Drumheller Feer, 1983). The increase in liver enzymes was fully reversible upon withdrawal of dexverapamil but necessitated cessation of treatment.

Dexverapamil did not increase the dose-limiting epirubicin toxicities, i.e. on the bone marrow and mucosa. Accordingly, epirubicin could be given at a similar dose intensity without and with dexverapamil. The mechanism underlying the slightly, albeit significantly, lower haemoglobin nadirs in the dexverapamil/epirubicin cohort of patients is unclear. The differences in haemoglobin nadirs were similar when comparing the head-to-head or all treatment cycles without and with dexverapamil. Thus, the observed decrease in haemoglobin nadirs was not a result of cumulative RBC toxicity by epirubicin. Verapamil itself is not known to produce RBC toxicity. Therefore, dexverapamil may have enhanced epirubicin toxicity on RBCs in some yet undefined way.

Dexverapamil significantly diminished the AUC of epirubicin, on average by 36%. This finding is consistent with previously reported data on the effect of dexverapamil on epirubicin AUC (Scheithauer et al, 1993) but is in contrast to the pattern of pharmacokinetic interaction usually observed between chemosensitizers and cytotoxic agents (Fisher et al, 1996). Dexverapamil significantly increased the volume of epirubicin distribution at steady state and the AUC of the non-toxic metabolites epirubicin-glucoronide and the 7-deoxy-aglycones (K Mross, personal communication). The mechanisms underlying these pharmacokinetic effects are not clear. It may be speculated that dexverapamil did alter tissue perfusion by virtue of its peripheral vascular activity. Furthermore, there may be direct interference by dexverapamil with particular steps in the hepatic metabolism of epirubicin. The anthracyclines doxorubicin and epirubicin appear to differ with respect to the type of pharmacokinetic interaction with verapamil. Oral dexverapamil has been recently reported to increase the steady-state concentration of doxorubicin by 50% (Wilson et al, 1995*a*). Similarly, racemic verapamil has been found to increase the peak plasma levels and terminal half-life of doxorubicin (Kerr et al, 1986), whereas it has shown no effect on epirubicin AUC (Mross et al, 1993*b*). Recently, oral dexverapamil has been found to increase the AUC of paclitaxel twofold (Berg et al, 1995), while it had no effect on the steady-state concentration of etoposide (Wilson et al, 1995*a*). With cyclosporin A and its analogue PSC 833, an increase in AUC and toxicity has been a consistent finding with any of the cytotoxic drugs tested so far (Lum et al, 1992; Bartlett et al, 1994; Boote et al, 1996). In contrast, the effects of other

© Cancer Research Campaign 1998

chemosensitizers on the pharmacokinetics and -dynamics of cytotoxic drugs appear to depend on the particular agents used in combination.

Verapamil and nor-verapamil have been previously found to be equipotent in reversing drug resistance in vitro (Merry et al, 1989). In the present study, the mean trough levels of dexverapamil and nor-dexverapamil combined were 2.7 μM, a concentration that is capable of reversing P-gp-associated MDR in experimental models. In our analyses of ex vivo P-gp-inhibitory activity, each serum sample from patients receiving dexverapamil proved capable of inhibiting P-gp function, with good statistical correlation between dexverapamil plasma levels and functional activity (Lehnert et al, 1996*b*). In the few plasma samples with dexverapamil levels of > 2.0 μM, the ratio of nor-dexverapamil to dexverapamil was <1, whereas the reverse was true in the 83% of samples with dexverapamil concentrations of <2.0 μM. This corroborates previous observations that the conversion of dexverapamil to nor-dexverapamil is a saturable process (Wilson et al, 1995*a*). Higher relative plasma concentrations of nor-dexverapamil have also been found in other clinical studies of dexverapamil (Scheithauer et al, 1993; Motzer et al, 1995) whereas, with racemic verapamil, the verapamil to norverapamil ratio has usually been ≥ 1 (Pennock et al, 1991; Mross et al, 1993*b*). Norverapamil is known to have only 20% of the cardiovascular activity of the parent compound, when using the racemic mixture of verapamil (Neugebauer, 1978). R-verapamil has been shown to have a five- to tenfold lower cardiac activity than does the S-enantiomer (Echizen et al, 1985), and thus nor-dexverapamil can be speculated to almost lack cardiac effects. Accordingly, a nor-dexverapamil to dexverapamil ratio of > 1 may be associated with diminished cardiac toxicity, without sacrificing MDR reversing potency.

For ethical reasons, we were not able to perform tumour biopsies for analysis of MDR1/P-gp expression. On theoretical grounds, such information appears to be important in chemosensitizer studies because, in the absence of functional P-gp expression, P-gp-inhibitors are not expected to overcome resistance at the cellular level. In a meta-analysis of all the original studies of MDR1/P-gp detection in clinical breast cancers, the proportion of MDR1/P-gp-positive tumours was around 40% (Trock et al, 1997). The results, however, have been highly variable (Goldstein et al, 1989; Merkel et al, 1989; Wishart et al, 1990; Sanfilippo et al, 1991; Verrelle et al, 1991; Wallner et al, 1991; Bates et al, 1995; Linn et al, 1995; Murren et al, 1996; Trock et al, 1997). One reason for the discrepant data seems to be the different sensitivity and specificity of the various detection methods used in these studies (Beck et al, 1996; Broxterman et al, 1996). Prior chemotherapy seems to significantly increase MDR1/P-gp expression in breast tumours and a significant association has been found between MDR1/P-gp positivity after chemotherapy and lack of response to the particular treatment (Trock et al, 1997). The biological and therapeutic implications of these data, however, remain to be determined (Kaye, 1997).

The observed ability of dexverapamil to overcome epirubicin refractoriness in patients with metastatic breast cancer appears to be encouraging. However, these data have to be considered as preliminary and need confirmation in larger studies. In particular, it remains to be determined in prospective randomized trials whether the addition of dexverapamil to epirubicin is able to improve progression-free and overall survival in patients with this disease. These reservations notwithstanding, the data from this study appear to contest two widely held notions, i.e. that the concept of enhancing chemotherapy activity by chemosensitizers may only function in haematological neoplasms and that an increase in the AUC and the toxicity of cytotoxic agents is a prerequisite for chemosensitizers to enhance anti-tumour activity.

### ACKNOWLEDGEMENTS

We thank D Schoebel, J Eiselstein and E Mogler-Drautz (all from Knoll AG, Ludwigshafen, Germany) for the analysis of dexverapamil and nor-dexverapamil plasma levels, statistical analyses and study monitoring respectively. We also want to thank J Greiner, I Worni and E Schernhammer for clinical patient monitoring and data management.

### REFERENCES

Baldini N, Scotlandi K, Barbanti-Brodano G, Manara MC, Maurici D, Bacci G, Bertoni F, Picci P, Sottilis, Campanacci M and Serra M (1995) Expression of P-glycoprotein in high-grade osteosarcoma in relation to clinical outcome. *N Engl J Med* **333**: 1380–1385

Bartlett NL, Lum BL, Fisher G, Brophy NA, Ehsan MN, Halsey J and Sikic BI (1994) Phase I trial of doxorubicin with cyclosporine as a modulator of multidrug resistance. *J Clin Oncol* **12**: 835–842

Bates SE, Meadows B, Goldspiel BR, Denicoff A, Tung BL, Tucker E, Steinberg S and Elwood LJ (1995) A pilot study of amiodarone with infusional doxorubicin or vinblastine in refractory breast cancer. *Cancer Chemother Pharmacol* **35**: 457–463

Beck WT, Grogan TM, Willman CL, Cordon-Cardo C, Parham DM, Kuttesch JF, Andreeff M, Bates SE, Berard CW, Boyett JM, Brophy NA, Broxterman HJ, Chan HSL, Dalton WS, Dietel M, Fojo AT, Gascoyne RD, Head D, Houghton PJ, Srivastava DK, Lehnert M, Leith CP, Paietta E, Pavelic ZP, Rimsza L, Roninson IB, Sikic BI, Twentyman PR, Warnke R and Weinstein R (1996) Methods to detect P-glycoprotein-associated multidrug resistance in patients' tumors: consensus recommendations. *Cancer Res* **56**: 3010–3020

Berg SL, Tolcher A, O'Shaughnessy JA, Denicoff AM, Noone M, Ognibene FP, Cowan KH and Balis FM (1995) Effect of R-verapamil on the pharmacokinetics of paclitaxel in women with breast cancer. *J Clin Oncol* **13**: 2039–2042

Bissett D, Kerr DJ, Cassidy J, Meredith P, Traugott U and Kaye SB (1991) Phase I and pharmacokinetic study of D-verapamil and doxorubicin. *Br J Cancer* **64**: 1168–1171

Boote DJ, Dennis IF, Twentyman PR, Osborne RJ, Laburte C, Hensel S, Smyth JF, Brampton MH and Bleehen NM (1996) Phase I study of etoposide with SDZ PSC 833 as a modulator of multidrug resistance in patients with cancer. *J Clin Oncol* **14**: 610–618

Brodsky SJ, Cutler SS, Weiner DA and Klein MD (1981) Hepatotoxicity due to treatment with verapamil. *Ann Intern Med* **94**: 490–491

Broxterman HJ, Lankelma J and Pinedo HM (1996) How to probe tumour samples for P-glycoprotein and multidrug resistance-associated protein. *Eur J Cancer* **32A**: 1024–1033

Budd GT, Bukowski RM, Lichtin A, Bauer L, Van Kirk P and Ganapathi R (1993) Phase II trial of doxorubicin and trifluoperazine in metastatic breast cancer. *Invest New Drugs* **11**: 75–79

Chan HSL, Thorner PS, Haddad G and Ling V (1990) Immunohistochemical detection of P-glycoprotein: prognostic correlation in soft tissue sarcoma of childhood. *J Clin Oncol* **8**: 689–704

Chan HSL, Haddad G, Thorner PS, DeBoer G, Lin YP, Ondrused N, Yeger H and Ling V (1991) P-glycoprotein expression as a predictor of the outcome of therapy for neuroblastoma. *N Engl J Med* **325**: 1608–1614

Echizen H, Brecht T, Niedergesaess S, Vogelsang B and Eichelbaum M (1985) The effect of dextro-levo- and racemic verapamil on atrio-ventricular conduction in humans. *Am Heart J* **109**: 210–217

Ferry DR, Traunecker H and Kerr DJ (1996) Clinical trials of P-glycoprotein reversal in solid tumours. *Eur J Cancer* **32A**: 1070–1081

Fisher GA, Lum BL, Hausdorff J and Sikic BI (1996) Pharmacological considerations in the modulation of multidrug resistance. *Eur J Cancer* **32A**: 1082–1088

Ford JM (1996) Experimental reversal of P-glycoprotein-mediated multidrug resistance by pharmacological chemosensitisers. *Eur J Cancer* **32A**: 991–1001

© Cancer Research Campaign 1998

Gehan EA and Schneiderman MA (1982) Experimental design of clinical trials. In *Cancer Medicine*, Holland JF and Frei E III. (eds), pp. 531–553. Lea and Febiger: Philadelphia

Goldstein LJ (1996) *MDR1* gene expression in solid tumours. *Eur J Cancer* **32A**: 1039–1050

Goldstein LJ, Galski H, Fojo A, Willingham M, Lai SL, Gazdar A, Pirker R, Green A, Crist W, Brodeur GM, Lieber M, Cossman J, Gottesman MM and Pastan I (1989) Expression of a multidrug resistance gene in human cancers. *J Natl Cancer Inst* **81**: 116–124

Gottesman MM and Pastan I (1993) Biochemistry of multidrug resistance mediated by the multidrug transporter. *Ann Rev Biochem* **62**: 385–427

Gruber A, Peterson C and Reizensten P (1988) D-verapamil and L-verapamil are equally effective in increasing vincristine accumulation in leukemic cells in vitro. *Int J Cancer* **41**: 224–226

Harapat SR and Kates RE (1980) High-performance liquid chromatographic analysis of verapamil. *J Chromatogr* **181**: 484–489

Hausmaninger H, Lehnert M, Steger G, Sevelda P, Tschurtschenthaler G, Hehenwarter W, Fridrik M, Samonigg H, Schiller L, Manfreda D, Haidinger R, Kienzer R and Kemmler G (1995) Randomized phase II study of epirubicin–vindesine versus mitoxantrone–vindesine in metastatic breast cancer. *Eur J Cancer* **31A**: 2169–2173

Hayward JL, Carbone PP, Heuson JC, Rubens ED, Kumaoka S and Segaloff A (1977) Assessment of response to therapy in advanced breast cancer. *Eur J Cancer* **13**: 89–94

Kaye SB (1997) Multidrug resistance in breast cancer – is the jury in yet? *J Natl Cancer Inst* **89**: 902–903

Kerr DJ, Graham J, Cummings J, Morrison JG, Thompson GG, Brodie MJ and Kaye SB (1986) The effect of verapamil on the pharmacokinetics of adriamycin. *Cancer Chemother Pharmacol* **18**: 239–242

Lehnert M (1996) Clinical multidrug resistance in cancer: a multifactorial problem. *Eur J Cancer* **32A**: 912–920

Lehnert M, De Giuli R and Twentyman PR (1996) A sensitive and rapid bioassay for analysis of P-glycoprotein-inhibiting activity of chemosensitizers in patient serum. *Clin Cancer Res* **2**: 403–410

Linn SC, Giaccone G, Van Diest PJ, Blokhuis WMD, Van Der Valk P, Van Kalken CK, Kuiper CM, Pinedo HM and Baak JPA (1995) Prognostic relevance of P-glycoprotein expression in breast cancer. *Ann Oncol* **6**: 679–685

List AF, Spier C, Greer J, Wolff S, Hutter J, Dorr R, Salmon SE, Futscher B, Baier M and Dalton W (1993) Phase I/II trial of cyclosporine as a chemotherapy-resistance modifier in acute leukemia. *J Clin Oncol* **11**: 1652–1660

Lum BL, Kraubisch S, Yahanda AM, Adler KM, Jew L, Ehsan MN, Brophy NA, Hlasey J, Gosland MP and Sikic BI (1992) Alteration of etoposide pharmacokinetics and pharmacodynamics by cyclosporine in a phase I trial to modulate multidrug resistance. *J Clin Oncol* **10**: 1635–1642

Maessen P, Mross K, Pinedo HM and Van Der Vijgh WJF (1987) Improved method for the determination of 4′-epidoxorubicin and seven metabolites in plasma by high-performance liquid chromatography. *J Chromatogr* **417**: 339–346

Marie J-P, Zhoud-S, Gurbuxani S, Legrand O and Zittoun R (1996) MDR1/P-glycoprotein in haematological neoplasms. *Eur J Cancer* **32A**: 1034–1038

Marschner N, Kreienberg R, Souchon R, Räth U, Eggeling B, Voigtmann R, Ruffert K, Schütte M, Ammon A, Kesztyüs T, Kaplan E and Nagel G (1994) Evaluation of the importance and relevance of dose intensity using epirubicin and cyclosphosphamide in metastatic breast cancer: interim analysis of a prospective randomized trial. *Semin Oncol* **21**: 10–16

Merkel DE, Fuqua SAW, Tandon AK, Hill SM, Buzdar AU and McGuire WL (1989) Electrophoretic analysis of 248 clinical breast cancer specimens for P-glycoprotein overexpression or gene amplification. *J Clin Oncol* **7**: 1129–1136

Merry S, Flanigan P, Schlick E, Freshney RI and Kaye SB (1989) Inherent adriamycin resistance in a murine tumour line: circumvention with verapamil and norverapamil. *Br J Cancer* **59**: 895–897

Miller AB, Hoogstraten B, Staquet M and Winkler A (1981) Reporting results of cancer treatment. *Cancer* **47**: 207–214

Miller TP, Grogan TM, Dalton WS, Spier CM, Scheper RJ and Salmon SE (1991) P-glycoprotein expression in malignant lymphoma and reversal of clinical drug resistance with chemotherapy plus high-dose verapamil. *J Clin Oncol* **9**: 17–24

Millward MJ, Cantwell BMJ, Munro NC, Robinson A, Corris PA and Harris AL (1993) Oral verapamil with chemotherapy for advanced non-small-cell lung cancer: a randomised study. *Br J Cancer* **67**: 1031–1035

Motzer RJ, Lyn P, Fischer P, Lianes P, Ngo RL, Cordon-Cardo C and O'Brien JP (1995) Phase I/II trial of dexverapamil plus vinblastine for patients with advanced renal cell carcinoma. *J Clin Oncol* **13**: 1958–1965

Mross K, Mayer U, Hamm K and Hossfeld DK (1990) High-performance liquid chromatography of iodo-doxorubicin and fluorescent metabolites in plasma samples. *J Chromatogr* **530**: 192–199

Mross K, Bohn C, Edler L, Jonat W, Queisser W, Heidemann E, Goebel M and Hossfeld DK (1993*a*) Randomized phase II study of single-agent epirubicin ± verapamil in patients with advanced metastatic breast cancer. *Ann Oncol* **4**: 45–50

Mross K, Hamm K and Hossfeld DK (1993*b*) Effects of verapamil on the pharmacokinetics and metabolism of epirubicin. *Cancer Chemother Pharmacol* **31**: 369–375

Murren JR, Durivage HJ, Buzaid AC, Reiss M, Flynn SD, Carter D and Hait WN (1996) Trifluoperazine as a modulator of multidrug resistance in refractory breast cancer. *Cancer Chemother Pharmacol* **38**: 65–70

Nash DT and Drumheller Feer T (1983) Hepatic injury possibly induced by verapamil. *JAMA* **249**: 395–396

Neugebauer G (1978) Comparative cardiovascular actions of verapamil and its major metabolites in the anaesthetised dog. *Cardiovas Res* **12**: 247–254

Ozols RF, Cunnion RE, Klecker RW, Hamilton TC, Ostchega Y, Perillo JE and Young RC (1987) Verapamil and Adriamycin in the treatment of drug-resistant overian cancer patients. *J Clin Oncol* **5**: 641–647

Pennock GD, Dalton WS, Roeske WR, Appleton AP, Mosley K, Plezia P, Miller TP and Salmon SE (1991) Systemic toxic effects associated with high-dose verapamil infusion and chemotherapy administration. *J Natl Cancer Inst* **83**: 105–110

Philip PA, Joel S, Monkman SC, Dolega-Ossowski E, Tonkjin K, Carmichael J, Idle JR and Harris AL (1992) A phase I study on the reversal of multidrug resistance (MDR) *in vivo*: nifedipine plus etoposide. *Br J Cancer* **65**: 267–270

Pinedo HM and Giaccone G (1995) P-glycoprotein – a marker of cancer-cell behavior. *N Engl J Med* **333**: 1417–1419

Pirker R, Keilhauer G, Raschak M, Lechner C and Ludwig H (1990) Reversal of multi-drug resistance in human KB cell lines by structural analogs of verapamil. *Int J Cancer* **45**: 916–919

Pirker R, Wallner J, Geissler K, Linkesch W, Haas OA, Bettelheim P, Hopfner M, Scherrer R, Valent P, Havelec L, Ludwig H and Lechner K (1991) MDR1 gene expression and treatment outcome in acute myeloid leukemia. *J Natl Cancer Inst* **83**: 708–712

Ries F and Dicato M (1991) Treatment of advanced and refractory breast cancer with doxorubicin, vincristine and continuous infusion of verapamil. A phase I–II clinical trial. *Med Oncol Tumor Pharmacother* **8**: 39–43

Salmon SE, Dalton WS, Grogan TM, Plezia P, Lehnert M, Roe DJ and Miller TM (1991) Multidrug-resistant myeloma, laboratory and clinical effects of verapamil as a chemosensitizer. *Blood* **78**: 44–50

Sanfilippo O, Ronchi E, De Marco C Di Fronzo G and Silvestrine R (1991) Expression of P-glycoprotein in breast cancer tissue and in vitro resistance to doxorubicin and vincristine. *Eur J Cancer* **27**: 155–158

Scheithauer W, Schenk T and Czeika M (1993) Pharmacokinetic interaction between epirubicin and the multidrug resistance reverting agent D-verapamil. *Br J Cancer* **68**: 8–9

Sikic BI (1993) Modulation of multidrug resistance: at the threshold. *J Clin Oncol* **11**: 1629–1635

Sonneveld P (1996) Reversal of multidrug resistance in acute myeloid leukaemia and other haematological malignancies. *Eur J Cancer* **32A**: 1062–1069

Sonneveld P, Durie BGM, Lokhorst HM, Marie JP, Solbu G, Suciu S, Zittoun R, Löwenberg B and Noter K (1992) Modulation of multidrug-resistant multiple myeloma with cyclosporin. *Lancet* **340**: 255–259

Trock BJ, Leonessa F and Clarke R (1997) Multidrug resistance in breast cancer: a meta-analysis of MDR1/gp170 expression and its possible functional significance. *J Natl Cancer Inst* **89**: 917–931

Van Kalken CK, Van Der Hoeven JJM, De Jong J, Giaccone G, Schuurhuis GJ, Maessen PA, Blokhuis WMD, Van Der Vijgh WJF and Pinedo HM (1991) Bepridil in combination with anthracyclines to reverse anthracycline resistance in cancer patients. *Eur J Cancer* **27**: 739–744

Verrelle P, Meissonnier F, Fonck Y, Feillel V, Dionet C, Kwiatkowski F, Plagne R and Chassagne J (1991) Clinical relevance of immunohistochemical detection of multidrug resistance P-glycoprotein in breast carcinoma. *J Natl Cancer Inst* **83**: 111–116

Wallner J, Depisch D, Hopfner M, Haider K, Spona J, Ludwig H and Pirker R (1991) MDR1 gene expression and prognostic factors in primary breast carcinomas. *Eur J Cancer* **27**: 1352–1355

Wilson WH, Jami-Dow C, Bryant G, Balis FM, Klecker RW, Bates SE, Chabner BA, Steinberg SM, Kohler DR and Wittes RE (1995*a*) Phase I and pharmacokinetic study of the multidrug resistance modulator dexverapamil with EPOCH chemotherapy. *J Clin Oncol* **13**: 1985–1994

© Cancer Research Campaign 1998

Wilson WH, Bates SE, Fojo A, Bryant G, Zhan Z, Regis J, Wittes RE, Jaffe ES, Steinberg SM, Herdt J and Chabner B (1995*b*) Controlled trial of dexverapamil, a modulator of multidrug resistance, in lymphomas refractory to EPOCH chemotherapy. *J Clin Oncol* **13**: 1995–2004

Wishart GC, Plumb JA, Going JJ, McNicol AM, McArdle CS, Tsuruo T and Kaye SB (1990) P-glycoprotein expression in primary breast cancer detected by immunocytochemistry with two monoclonal antibodies. *Br J Cancer* **62**: 758–761

Wishart GC, Plumb JA, Morrison JG, Hamilton TG and Kaye SB (1992) Adequate tumor quinidine levels for multidrug resistance modulation can be achieved in vivo. *Eur J Cancer* **28**: 28–31

Wishart GC, Bissett D, Paul J, Jodrell D, Harnett A, Habeshaw T, Kerr DJ, Macham MA, Soukop M, Leonard RCF, Knepil J and Kaye SB (1994) Quinidine as a resistance modulator of epirubicin in advanced breast cancer: mature results of a placebo-controlled randomized trial. *J Clin Oncol* **12**: 1771–1777

© Cancer Research Campaign 1998